# An Open-Label Uncontrolled, Multicenter Study for the Evaluation of the Efficacy and Safety of the Dermal Filler Princess VOLUME in the Treatment of Nasolabial Folds

**DOI:** 10.1155/2015/195328

**Published:** 2015-03-03

**Authors:** Daisy Kopera, Michael Palatin, Rolf Bartsch, Katrin Bartsch, Maria O'Rourke, Sonja Höller, Renate R. Baumgartner, Martin Prinz

**Affiliations:** ^1^Department of Dermatology and Venereology, Medical University of Graz, Auenbruggerplatz 8, 8036 Graz, Austria; ^2^Praxis Dr. Palatin, Rotenturmstrasse 1, 1010 Vienna, Austria; ^3^Institut für Plastische Chirurgie Wien 19 GmbH, Sieveringer Straße 36, 1190 Wien, Austria; ^4^Clinical Development, CROMA PHARMA GmbH, Industriezeile 6, 2100 Leobendorf, Austria

## Abstract

The dermal filler Princess VOLUME is a highly cross-linked, viscoelastic hyaluronic acid injectable gel implant used for aesthetic treatment. To evaluate the efficacy and safety of Princess VOLUME in the treatment of nasolabial folds, an open-label uncontrolled, multicenter study was conducted. Forty-eight subjects were recruited who had moderate to deep wrinkles, according to the Modified Fitzpatrick Wrinkle Scale (MFWS). Subjects received Princess VOLUME in both nasolabial folds at Day 0. Nasolabial fold severity was evaluated at 30, 90, 180, and 270 days after treatment, using the MFWS and the Global Aesthetic Improvement Scale (GAIS). Adverse events and treatment site reactions were recorded. Among the 48 subjects, 93.8% were female with a median age of 52 years. There were significant improvements (*P* < 0.0001) in the MFWS scores at 30, 180, and 270 days after treatment compared with those at baseline, with a mean decrease of 1.484 (±0.408), 1.309 (±0.373), and 1.223 (±0.401), respectively; hence the primary endpoint was achieved and clinical efficacy demonstrated. Princess VOLUME was well tolerated, and most adverse events were injection site reactions of mild to moderate severity. Subject satisfaction (97.9%), subject recommendation of the treatment (93.6%), and investigators GAIS scores (97.9% improvement) were high.

## 1. Introduction

The facial signs of aging are characterized by the formation of wrinkles and folds, which result from the loss of bone mass and soft tissue volume, the redistribution of fat, and decreased skin elasticity and thickness [1–4]. With the natural process of aging, the endogenous hyaluronic acid (HA) is reduced, resulting in a less hydrated and consequently less elastic skin [5]. In the midface, furrowing and flattening of the central mid cheek occur, with displacement medially exaggerating the depth of the nasolabial folds [6–9].

Dermal fillers serve as one of the most common and useful treatments for wrinkles and folds and appeal to the growing population keen to reverse the signs of aging [9]. Over the past two decades, soft tissue augmentation using injectable fillers has become a standard clinical approach for correcting age-related facial defects [9]. Among these, HA-based fillers appear to be ideal due to their low immunogenic potential and relatively long-lasting effect. Moreover, due to their favorable risk/benefit profile, HA products are the most widely used dermal fillers in Europe and USA. HA is a naturally occurring glycosaminoglycan which exhibits no species or tissue specificity and is an essential component of the extracellular matrix in adult tissue [3, 10–12]. In the skin, it is located among the collagen fibers and has a hydrophilic capability, playing a critical role in the maintenance and regulation of hydration within tissues and contributing to skin turgor [3, 5, 13, 14].

A wide range of HA-based dermal fillers are currently available which differ in terms of source material, concentration, method, and degree of cross-linking, viscosity, consistency, stiffness, and injection force [15]. Chemical modification of HA by cross-linking has been reported to improve consistency and increase the intradermal residence time of the filler [16, 17]. Princess VOLUME filler is created by using a unique SMART technology (Supreme Monophasic and Reticulated Technology) based on biofermentative HA cross-linking with 1,4-butanediol diglycidyl ether (BDDE), which enables a highly dense tridimensional matrix. Compared with native HA, the cross-linked HA gel is considerably more resistant to enzymatic and free radical breakdown, leading to increased tissue residence time. The product has an excellent safety profile. Biocompatibility testing* in vivo* has confirmed Princess VOLUME to be noncytotoxic, nonirritant, nontoxic, nonsensitizing, and nonpyrogenic. Furthermore, the limit of BDDE residuals in the finished product is <2 ppm, which is one of the lowest reported values in dermal fillers on the market.

The aim of this open-label uncontrolled, multicenter study was to evaluate the safety and efficacy of the dermal filler Princess VOLUME in the treatment of nasolabial folds in female and male subjects aged between 30 and 65 years over a period of 9 months.

## 2. Materials and Methods

### 2.1. Materials

The dermal filler Princess VOLUME, based on biofermentative HA, carries the European Conformity mark (CE) and has been on the European market since 2009. Princess VOLUME is a highly cross-linked, viscoelastic HA injectable gel implant at a concentration of 23 mg/mL in a physiological buffer.

The volume injected was chosen by the investigator and depended on the depth of wrinkle to be corrected. If full correction was not achieved after the initial treatment, a single touch-up treatment was permitted at Day 14.

### 2.2. Patient Selection and Clinical Study

This open-label uncontrolled, multicenter study for the evaluation of the efficacy and safety of the dermal filler Princess VOLUME in nasolabial folds was conducted between 9 April 2013 and 1 July 2014 at three sites in Austria (EUDAMED Study number CIV-AT-13-04-010527).

The criteria for inclusion in the study were male or female subjects between 30 and 65 years with a score of at least 2 according to the Modified Fitzpatrick Wrinkle Scale (MFWS [18]). Subjects had to abstain from any cosmetic or surgical procedures (including botox injection) in the treatment area during the clinical investigation, subjects had to be free of diseases that could interfere in cutaneous aging evaluation, and subjects had to refrain from excessive weight gain or loss during the investigation period.

Subjects were excluded from the study for any of the following reasons: if they had previously received permanent implants; if they had any facial surgery or implantation of dermal fillers in the nasolabial region within the previous 24 months before enrollment; if they underwent procedures such as laser therapy, chemical peeling, dermabrasion, or botulinum toxin injection within 12 months before enrollment; if they had dermatological problems (cutaneous lesions, hypertrophic scars, or a tendency to keloid formation); if they had systemic diseases (diabetes mellitus, connective tissue diseases, or uncontrolled systemic diseases); if they had immune system disorders (autoimmune disease, HIV positive status, history of immune system degradation, or recurrent herpes simplex); if they had previously shown allergies to HA or cosmetic fillers; if they were pregnant, lactating, or unwilling to exercise birth control; if they received any other investigational treatment or had participated in another clinical study within 30 days prior to study enrollment or had any medical condition or taken any medication that in the investigator's judgment would prohibit inclusion in the study.

Before participation in the study, subjects received patient information and signed and dated the informed consent form. The study was conducted in accordance with International Standards Organization 14155:2011: Clinical investigation of medical devices for human subjects-Good clinical practice (GCP), the principles of the Helsinki Declaration, and the applicable sections of the national medical device law. Ethics committee approval was obtained before study initiation (from the Medical University of Graz, the City of Vienna and the county of Styria).

Before treatment, subjects received a brief general examination including medical history and survey of current medication. Pretreatment baseline photographs of the right and left nasolabial folds (NLFs) were taken for each subject. Eligible subjects received injections of Princess VOLUME into both nasolabial folds at baseline (Day 0). Follow-up clinical visits for assessment of safety and efficacy were performed at Days 14, 30, 90, 180, and 270. Touch-up treatment was performed on Day 14, if considered necessary by the investigator. Photographic documentation of the nasolabial region was taken on all visits.

### 2.3. Efficacy Assessment

Clinical efficacy was assessed by the investigator on 30, 90, 180, and 270 days after baseline, using the Modified Fitzpatrick Wrinkle Scale [18] and the Global Aesthetic Improvement Scale (GAIS). Subject's assessment of efficacy was evaluated through subject questionnaires.

The MFWS is a validated 7-point rating scale (ranging from 0 = no wrinkle to 3 = deep wrinkle, Table 1) used to quantify the results obtained from the treatment of nasolabial folds on the basis of photographic images [18]. Wrinkle depth was assessed by the investigator using four reference photographs, rather than on physical measurement.

The Global Aesthetic Improvement Scale (GAIS) is a 5-point scale rating global aesthetic improvement in appearance, compared to pretreatment, as judged by the investigator. The rating categories were “worse,” “no change,” “improved,” “much improved,” and “very much improved.” GAIS was measured for each nasolabial fold at baseline and during follow-up, with results compared to the baseline pretreatment photographs.

To evaluate subject satisfaction, subjects were asked to complete a questionnaire. At Days 30, 90, 180, and 270 subjects were asked how they would judge their change of appearance after treatment using a 5-point scale [very much improved (1), much improved (2), slightly improved (3), no change (4), or worsened (5)]. At Days 30 and 270, subjects were asked to indicate their degree of satisfaction with the treatment on a three-point scale (very satisfied, satisfied, or not satisfied); subjects were also asked whether they would recommend the treatment further to friends and acquaintances (yes, perhaps, or no). Subjects completed their self-evaluation independently, prior to the investigator's evaluation.

### 2.4. Safety Assessment

Evaluation of safety and tolerability of the study product was based on spontaneous reporting of adverse events by the subjects, as well as evaluation of the subject's general health at each study visit. The treatment area was assessed for local reactions by the investigators at scheduled visits.

### 2.5. Endpoints

The primary efficacy endpoint was the absolute change in MFWS from Day 0 to Day 180. The secondary efficacy endpoints included the change in MFWS from Day 0 to Days 30, 90, and 270, the percentage of subjects with an improvement of at least 0.5 on the MFWS at Days 30, 90, 180, and 270, improvement in Global Aesthetic Improvement Scale (GAIS) at Days 30, 90, 180, and 270, and subject satisfaction at Days 30, 90, 180, and 270. For the evaluation of the safety endpoint, the frequency and severity of adverse events (AEs) were documented at each study visit.

### 2.6. Statistical Analysis

The change in MFWS from Day 0 to Day 180 was analyzed using a Wilcoxon signed rank test with a 2-sided significance level of 5%, using the mean of the two measurements of both nasolabial folds (right and left). The primary efficacy analysis was based on the safety data set and was considered confirmatory. The per protocol analysis set was used for an explanatory analysis.

## 3. Results

### 3.1. Subjects

A total of 48 subjects were enrolled in the study at three selected sites. Forty-seven subjects completed the study, and one subject was lost to follow-up after Day 14. Six subjects were excluded from the per protocol analysis due to protocol deviations (two subjects violated a single inclusion/exclusion criterion, three subjects had time window violations, and one subject discontinued the study prematurely). The majority of subjects in the study were female (*n* = 45; 93.8%). The age of the study population ranged from 31 to 65 years, with a median age of 52 years. All subjects were Austrian and Caucasian.

### 3.2. Dosing and Administration

The mean injected volume of Princess VOLUME administered to each nasolabial fold was 0.8 mL. A total of 9 subjects (18.8%) received touch-up treatment on Day 14 and the mean injected volume for touch-up treatment to each nasolabial fold was 0.3 mL. Of the 48 subjects who were treated with the investigational device, 16 (33.3%) received anesthetic cream for device application on Day 0. No rescue medication (hyaluronidase) was administered during the study.

### 3.3. Efficacy

#### 3.3.1. Improvement in Wrinkle Severity according to Modified Fitzpatrick Wrinkle Scale

At baseline, prior to treatment, the severity of the nasolabial folds was assessed (MFWS) by the investigator as Grade 2 (moderate wrinkles) in 58.3% of subjects, Grade 2.5 (prominent and visible wrinkles) in 36.5% of subjects, and Grade 3 (deep wrinkles) in 5.2% of subjects (Table 2, mean of both values for right and left NLFs).

**Figure 1:**
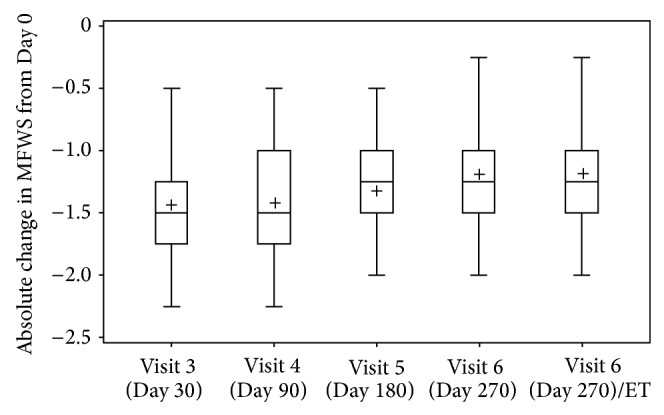
Absolute change in Modified Fitzpatrick Wrinkle Scale score from baseline for each visit. The mean of both measurements (right/left nasolabial fold) is used. Absolute change is calculated as follows: result for respective study visit minus Day 0 result. Box plots: Box displaying lower and upper quartile; median value is displayed by horizontal line, mean by “+,” and endpoints of upper and lower whiskers display maximum and minimum values.

The absolute change in MFWS score from baseline for each of the visits is shown in Figure 1. At Day 30 the mean NLF severity was reduced by 1.484 (±0.408), which was a significant improvement in MFWS compared to baseline (*P* value < 0.0001, 2-sided Wilcoxon signed rank test). At Day 180 the mean improvement in MFWS was 1.309 (±0.373), corresponding to a significant improvement compared to baseline (*P* value < 0.0001, 2-sided Wilcoxon signed rank test); thus the primary endpoint was achieved and clinical efficacy was demonstrated. At the last study visit on Day 270, the mean improvement in MFWS was 1.223 (±0.401), which was a significant improvement compared to baseline (*P* value < 0.0001, 2-sided Wilcoxon signed rank test).

**Figure 2:**
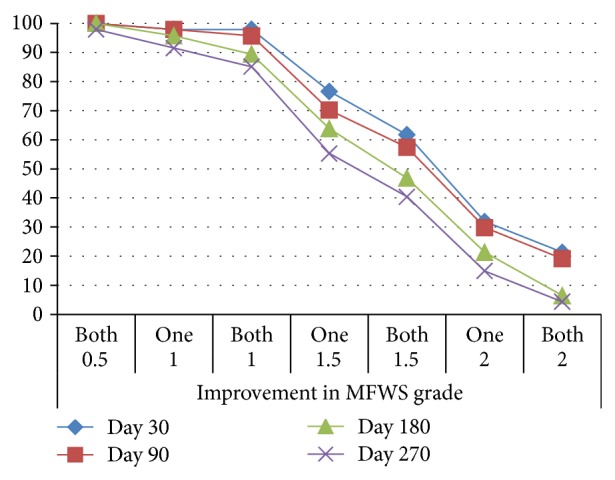
Percentage of subjects with an improvement in Modified Fitzpatrick Wrinkle Scale in one or both nasolabial folds at each study visit. MFWS: Modified Fitzpatrick Wrinkle Scale; grade characteristics are defined in Table 1. One: one nasolabial fold. Both: both nasolabial folds.

The percentage of subjects with an improvement in MFWS in one or both nasolabial folds over time is shown in Figure 2. An improvement in MFWS of 1 grade in both nasolabial folds was judged by investigators in 97.9% of subjects at Day 30, in 95.7% of subjects at Day 90, in 89.4% of subjects at Day 180, and in 85.1% of subjects at Day 270 (Figure 2). An improvement in MFWS of 1.5 grades in at least one nasolabial fold was evaluated by the investigators in 76.6% of subjects at Day 30, in 70.2% of subjects at Day 90, in 63.8% of subjects at Day 180, and in 55.3% of subjects at Day 270 (Figure 2). The median MFWS grade improved from 2.0 at Day 0 to 1.0 at Day 14 and was maintained at all later visits up to Day 270.

#### 3.3.2. Global Aesthetic Improvement

**Figure 3:**
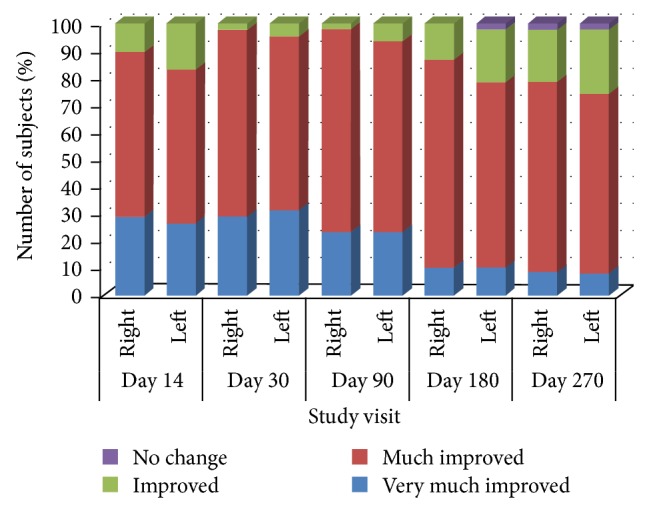
Global Aesthetic Improvement Scale assessments at each study visit compared to baseline. Designations right and left: corresponding to right and left nasolabial folds.

On the basis of investigator-evaluated GAIS scores, nasolabial fold appearance had improved in 100% of subjects at Day 14, Day 30, and Day 90 and in 97.9% of subjects at Days 180 and 270. For the majority of subjects, investigator judged the nasolabial fold appearance based on GAIS as “much improved” at all visits compared to Day 0 (Figure 3).

### 3.4. Subject Satisfaction

At Day 30 all of the subjects who took part in the study were satisfied with the treatment; 89.4% were very satisfied and 10.6% were satisfied (Table 3). The majority of subjects (97.9%) would recommend the treatment to others (Table 3). The assessments of “satisfaction with treatment” and “recommendation of treatment” were maintained by the majority of subjects for the duration of the study from Day 30 to Day 270 (Table 3). The subjects' judgment of appearance after treatment changed with time; however, by termination of the study on Day 270, the majority of subjects still considered their appearance improved or very much improved.

### 3.5. Safety

A total of 24 AEs were reported in 15 (31.3%) subjects (Table 4). Fourteen AEs reported by 14 subjects had a possible or definite relationship to the investigational device and were classified as adverse device effects (ADEs). An SAE, uterine polyp, was reported in a single subject and was considered unrelated to the investigational device by the investigator. The most frequently reported AEs were injection site hematoma and injection site swelling (reported by twelve and two subjects, resp.) and were the only AEs reported by more than one subject. The majority of AEs were mild (*n* = 16; 66.7%), 6 (25.0%) were moderate in severity, and 2 AEs (8.3%) were severe (trigger finger and uterine polyp). The majority of AEs had resolved by study completion and only two unrelated AEs (tendovaginitis and herpes labialis) were ongoing at the end of the study.

## 4. Discussion

This study enrolled 48 subjects who were predominantly women aged 31 to 65 years with moderate to deep wrinkles according to the Modified Fitzpatrick Wrinkle Scale. The objective of the study was to assess the safety and efficacy of Princess VOLUME in achieving significant correction in nasolabial folds and to assess whether this initial correction would persist over the nine-month duration of the study.

A variety of different scales have been used in evaluating efficacy in aesthetic clinical studies, including the wrinkle severity rating scale [19] and the midface volume deficiency scale [20]; these are 5-point scales to evaluate wrinkle fold severity against a set of reference photographs of nasolabial folds and have been validated. In this study, efficacy was evaluated by the investigator at Days 30, 90, 180, and 270 using the Modified Fitzpatrick Wrinkle Scale (MFWS [18]). The MFWS is a 7-point scale (Grade 0 to Grade 3) which, in addition to reference photographs, includes a series of clear and concise descriptions for each class, reflecting the deeper wrinkling and groove formation typical of the nasolabial fold [18]. The addition of a series of clear and concise descriptions for each class has resulted in greater precision of the MFWS and it has been proven to be a reliable wrinkle scoring system for nasolabial skin folds [18]. The MFWS has been validated [18] and before onset of the study, adequate training and instruction were provided at each site prior to use, in order to ensure proper assessment and grading.

At Day 30 after a single treatment with Princess VOLUME, there was a significant improvement in MFWS compared to baseline in almost all subjects, consistent with a significant reduction in nasolabial fold severity according to the MFWS scale. The treatment effect was maintained throughout the study period, with an improvement in MFWS (evaluated by the investigators) of up to 1 grade in both nasolabial folds reported in 89.4% and 85.1% of subjects at Day 180 and Day 270, respectively. The degree of improvement was less pronounced at later evaluation dates, which is consistent with reports that the effect of cross-linked HA usually lasts about 4 to 12 months [21]; however at Day 180 and Day 270 there was still a significant improvement in MFWS compared to baseline (*P* value < 0.0001). Moreover, by Day 270 the number of subjects who would have required a second injection, based on the severity of NLF used in the inclusion criteria, was <5% (1 out of 47) suggesting a long duration of action.

Investigator GAIS results confirmed these findings, with the nasolabial fold appearance judged by the investigator as “much improved” or “very much improved” for the majority of subjects at all visits compared to Day 0, showing 97.9% GAIS improvement at study end (Day 270). HA fillers provide temporary correction of the area treated, with eventual resorption of the material and a presumed return to the patient's pretreatment state. Other studies have reported that repeated treatments performed at 6–9 months (while some correction remains), has several advantages, namely, that the patient can benefit from a prolonged result using a fraction of the initial injection volume and also there are less dramatic changes in the appearance over time, resulting in a more natural appearance and less noticeable treatment effects [22]. On the basis of the MFWS and GAIS data, efficacy was clearly demonstrated up to 270 days after treatment with Princess VOLUME, suggesting that repeated treatment at 6 months is not necessary.

Subject ratings demonstrated high and consistent satisfaction throughout the duration of the study. Subject assessments were obtained independently of investigator assessments; however there was excellent agreement between the subject's assessment of improvement and the investigator-evaluated GAIS scores. Subject's recommendation of treatment at the final visit on Day 270 still demonstrated high satisfaction (97.9%) and readiness for further treatment (93.6%). The majority of the subjects completed the study and only 1 subject (2.1%) was lost to follow-up after Day 14, which is consistent with the high satisfaction rate reported by the subjects at each of the scheduled study visits.

Princess VOLUME was well tolerated, with most adverse events being injection site reactions of mild to moderate severity. Hematoma in the treated area was the most frequently reported ADE and all resolved without intervention. The single SAE reported in one subject (uterine polyp) was considered to be unrelated to the investigational device and resolved before completion of the study.

This study demonstrates the safety and efficacy of the dermal filler Princess VOLUME in the treatment of nasolabial folds in 47 subjects over a period of 9 months. A successive clinical study currently being conducted aims to compare the safety and efficacy of Princess VOLUME with a comparator product for the treatment of nasolabial folds.

## 5. Conclusions

There were significant improvements (*P* < 0.0001) in the MFWS scores on 30, 180, and 270 days compared with those at baseline, consistent with a significant reduction in nasolabial fold severity according to the MFWS scale.

The treatment effect was maintained throughout the study period, with an improvement in MFWS (evaluated by the investigators) of up to 1 grade in both nasolabial folds reported in 89.4% and 85.1% of subjects at Day 180 and Day 270, respectively.

On the basis of GAIS results, the nasolabial fold appearance was judged by the investigator as “much improved” or “very much improved” for the majority of subjects at all visits compared to Day 0, with 97.9% GAIS improvement at the end of the study (Day 270).

Subject satisfaction was high. At Day 30 all subjects who took part in the study were satisfied with the treatment; the majority (89.4%) were very satisfied. At the final visit on Day 270, 97.9% of subjects were satisfied with the treatment; the majority (80.9%) were still very satisfied.

Subject recommendation of the treatment was high, at 96.3%.

Princess VOLUME was well tolerated, with most adverse events being injection site reactions of mild to moderate severity.

## Figures and Tables

**Table 1:** Modified Fitzpatrick Wrinkle Scale grade definitions^*^.

Grade	Definition
0	No wrinkle: no visible wrinkle, continuous skin line
0.5	Very shallow yet visible wrinkle
1	Fine wrinkle: visible wrinkle and slight indentation
1.5	Visible wrinkle and clear indentation, <1 mm wrinkle depth
2	Moderate wrinkle: clear visible wrinkle 1-2 mm wrinkle
2.5	Prominent and visible wrinkle: more than 2 mm and less than 3 mm wrinkle depth
3	Deep wrinkle: deep and furrow wrinkle; more than 3 mm wrinkle depth

^*^As outlined in [18]. Wrinkle depth is based on assessors' estimation rather than physical measurement.

**Table 2:** MFWS grades at each study time point.

Day	NLF	Number (%)^a^ of subjects with MFWS grade						
		0	0.5	1	1.5	2	2.5	3
Day 0 (*N* = 48)	Right	—	—	—	—	30 (62.5)	16 (33.3)	2 (4.2)
	Left	—	—	—	—	26 (54.2)	19 (39.6)	3 (6.3)

Day 14 (*N* = 48)	Right	4 (8.3)	18 (37.5)	19 (39.6)	7 (14.6)	—	—	—
	Left	6 (12.5)	11 (22.9)	24 (50.0)	7 (14.6)	—	—	—

Day 30 (*N* = 47)^b^	Right	4 (8.5)	18 (38.3)	22 (46.8)	3 (6.4)	—	—	—
	Left	5 (10.6)	16 (34.0)	24 (51.1)	2 (4.3)	—	—	—

Day 90 (*N* = 47)^b^	Right	4 (8.5)	15 (31.9)	25 (53.2)	3 (6.4)	—	—	—
	Left	4 (8.5)	13 (27.7)	27 (57.4)	3 (6.4)	—	—	—

Day 180 (*N* = 47)^b^	Right	1 (2.1)	14 (29.8)	25 (53.2)	6 (12.8)	1 (2.1)	—	—
	Left	1 (2.1)	13 (27.7)	26 (55.3)	5 (10.6)	2 (4.3)	—	—

Day 270 (*N* = 47)^b^	Right	—	14 (29.8)	20 (42.6)	12 (25.5)	—	—	1 (2.1)
	Left	—	13 (27.7)	23 (48.9)	10 (21.3)	—	1 (2.1)	—

Grade 0 = no wrinkle; Grade 0.5 = very shallow yet visible wrinkle; Grade 1 = fine wrinkle; Grade 1.5 = visible wrinkle and clear indentation; Grade 2 = moderate wrinkle; Grade 2.5 = prominent and visible wrinkle; Grade 3 = deep wrinkle

^a^Percentages are based on the number of subjects with available data.

^b^One subject was lost to follow-up after Day 14.

Safety analysis data set: SAF.

MFWS = Modified Fitzpatrick Wrinkle Scale, *N* = number of subjects with available data, and NLF = nasolabial fold.

**Table 3:** Subject satisfaction.

Satisfaction	Number (%)^a^ of subjects			
	Day 30	Day 90	Day 180	Day 270
Q1: appearance after treatment (*N* = 47)				
Very much Improved	16 (34.0)	6 (12.8)	2 (4.3)	5 (10.6)
Much improved	27 (57.4)	37 (78.7)	30 (63.8)	25 (53.2)
Slightly improved	4 (8.5)	4 (8.5)	15 (31.9)	15 (31.9)
No change	—	—	—	2 (4.3)

Q2: satisfaction with treatment (*N* = 47)				
Very satisfied	42 (89.4)	NA	NA	38 (80.9)
Satisfied	5 (10.6)	NA	NA	8 (17.0)
Not satisfied	—	NA	NA	1 (2.1)

Q3: recommendation of treatment (*N* = 47)				
Yes	46 (97.9)	NA	NA	44 (93.6)
Perhaps	1 (2.1)	NA	NA	3 (6.4)

^a^The percentage is based on the number of subjects with available data. Data: safety analysis set.

*N* = number of subjects with available data, NA = not assessed, and Q = question.

**Table 4:** Adverse events by system organ class (*N* = 48).

System organ class (MedDRA) Preferred term	Number (%) of subjects^a^	
General disorders and administration site conditions	**14**	**(29.2)**
Influenza-like illness	1	(2.1)
Injection site hematoma	12	(25.0)
Injection site swelling	2	(4.2)
Infections and infestations	**3**	(6.3)
Acute tonsillitis	1	(2.1)
Bronchitis	1	(2.1)
Nasopharyngitis	1	(2.1)
Oral herpes	1	(2.1)
Musculoskeletal and connective tissue disorders	**1**	(2.1)
Tenosynovitis	1	(2.1)
Trigger finger	1	(2.1)
Reproductive system and breast disorders	**1**	(2.1)
Uterine polyp	1	(2.1)
Vaginal hemorrhage	1	(2.1)
Total	**15**	**(31.3)**

^a^Percentages are based on the total number of subjects in the safety analysis data.

MedDRA = Medical Dictionary for Regulatory Activities, *N* = number of subjects.

## Abbreviations

AE: Adverse event
ADE: Adverse device effect
GAIS: Global Aesthetic Improvement Scale
MFWS: Modified Fitzpatrick Wrinkle Scale
NLF: Nasolabial fold.

## References

[1] Lowe N. J., Maxwell C. A., Patnaik R. (2005). Adverse reactions to dermal fillers: review. *Dermatologic Surgery*.

[2] Smith K. C. (2008). Reversible vs. nonreversible fillers in facial aesthetics: concerns and considerations. *Dermatology Online Journal*.

[3] John H. E., Price R. D. (2009). Perspectives in the selection of hyaluronic acid fillers for facial wrinkles and aging skin. *Patient Preference and Adherence*.

[4] Donofrio L. M. (2000). Fat distribution: a morphologic study of the aging face. *Dermatologic Surgery*.

[5] Allemann I. B., Baumann L. (2008). Hyaluronic acid gel (Juvéderm) preparations in the treatment of facial wrinkles and folds. *Clinical Interventions in Aging*.

[6] Monheit G. D., Thomas J. A., Murphy D. K. Photographic documentation from a double-blind, randomized, multicenter study comparing new hyaluronic acid-based fillers vs. crosslinked bovine collagen.

[7] Coleman S. R., Grover R. (2006). The anatomy of the aging face: volume loss and changes in 3-dimensional topography. *Aesthetic Surgery Journal*.

[8] Mendelson B. C., Jacobson S. R. (2008). Surgical anatomy of the midcheek: facial layers, spaces, and the midcheek segments. *Clinics in Plastic Surgery*.

[9] Raspaldo H. (2008). Volumizing effect of a new hyaluronic acid sub-dermal facial filler: a retrospective analysis based on 102 cases. *Journal of Cosmetic and Laser Therapy*.

[10] Rao J., Chi G. C., Goldman M. P. (2005). Clinical comparison between two hyaluronic acid-derived fillers in the treatment of nasolabial folds: hylaform versus restylane. *Dermatologic Surgery*.

[11] Vedamurthy M. (2004). Soft tissue augmentation—use of hyaluronic acid as dermal filler. *Indian Journal of Dermatology, Venereology and Leprology*.

[12] Carruthers J. D. A., Glogau R. G., Blitzer A. (2008). Advances in facial rejuvenation: botulinum toxin type A, hyaluronic acid dermal fillers, and combination therapies—consensus recommendations. *Plastic & Reconstructive Surgery*.

[13] Comper W. D., Laurent T. C. (1978). Physiological function of connective tissue polysaccharides. *Physiological Reviews*.

[14] Tezel A., Fredrickson G. H. (2008). The science of hyaluronic acid dermal fillers. *Journal of Cosmetic and Laser Therapy*.

[15] Landau M. (2011). Examining the safety and efficacy of the non-animal cross-linked hyaluronic acid. *International Journal of Aesthetic and Antiaging Medicine*.

[16] Larsen N. E., Pollak C. T., Reiner K., Leshchiner E., Balazs E. A. (1993). Hylan gel biomaterial: dermal and immunologic compatibility. *Journal of Biomedical Materials Research*.

[17] Friedman P. M., Mafong E. A., Kauvar A. N. B., Geronemus R. G. (2002). Safety data of injectable nonanimal stabilized hyaluronic acid gel for soft tissue augmentation. *Dermatologic Surgery*.

[18] Shoshani D., Markovitz E., Monstrey S. J., Narins D. J. (2008). The modified Fitzpatrick Wrinkle Scale: a clinical validated measurement tool for nasolabial wrinkle severity assessment. *Dermatologic Surgery*.

[19] Day D. J., Littler C. M., Swift R. W., Gottlieb S. (2004). The wrinkle severity rating scale: a validation study. *The American Journal of Clinical Dermatology*.

[20] Callan P., Goodman G. J., Carlisle I. (2013). Efficacy and safety of a hyaluronic acid filler in subjects treated for correction of midface volume deficiency: a 24 month study. *Clinical, Cosmetic and Investigational Dermatology*.

[21] Carruthers A., Carruthers J. (2007). Non-animal-based hyaluronic acid fillers: scientific and technical considerations. *Plastic and Reconstructive Surgery*.

[22] Smith S. R., Jones D., Thomas J. A., Murphy D. K., Beddingfield F. C. (2010). Duration of wrinkle correction following repeat treatment with Juvéderm hyaluronic acid fillers. *Archives of Dermatological Research*.

